# Bilateral total knee arthroplasty with modified primary components in the management of neuropathic arthropathy related to chronic pancreatitis: a case with 5-year follow-up

**DOI:** 10.1007/s00590-012-1017-9

**Published:** 2012-06-13

**Authors:** Semih Aydogdu, Murat Ozturk, Murat Sezak, Hakkı Sur

**Affiliations:** 1 Department of Orthopaedic Surgery, School of Medicine Hospital, Ege University, Bornova, Izmir, 35100 Turkey; 2 Department of Pathology, Ege University Hospital, Izmir, Turkey

**Keywords:** Knee, Neuropathic arthropathy, Charcot joint, Arthroplasty

## Abstract

Although neuropathic arthropathy of the foot and ankle joints in diabetes is well known, the involvement of the knee joint is rare. Then, the management, particularly the use of arthroplasty, is a matter of debate. We report a 51-year-old man with neuroarthropathic involvement of both knees related to the chronic pancreatitis treated by modified primary total knee arthroplasty components using augments and extension stems only on the tibial side. Our 5-year observations revealed that although the replacement surgery in neuroarthropathy is associated with high rate of complications, it is highly effective in avoiding progressive functional disability and durable.

## Introduction

Neuropathic arthropathy is a great therapeutic challenge and there is no one solution that provides a durable and functional result. The absence of any self-limiting factor, such as protective pain sensation, can cause a progressive course and may result in a “highly difficult to treat” status. Therefore, timely management is of the most importance. Replacement procedures are reputed with high complication rates and have been considered by some in those cases as a contraindication [1, 2]. This case report represents a patient with neuropathic arthropathy of both knees and treated by staged total knee arthroplasty.

### Case report

A 51-year-old male patient with recent-onset bilateral mild-to-moderate knee pain and progressive deformity just below the knee joints was referred to us during his in-patient treatment at the department of gastroenterology of our institution. The patient had a history of chronic pancreatitis related to the excessive alcohol consumption over the last 15 years. His pancreatitis was complicated with type II diabetes that required insulin treatment for the last 4 years. He had chronic diarrhea for the last 3 years with up to 7–8 defecations per day. The patient had no history of therapeutic steroid use.

**Fig. 1:**
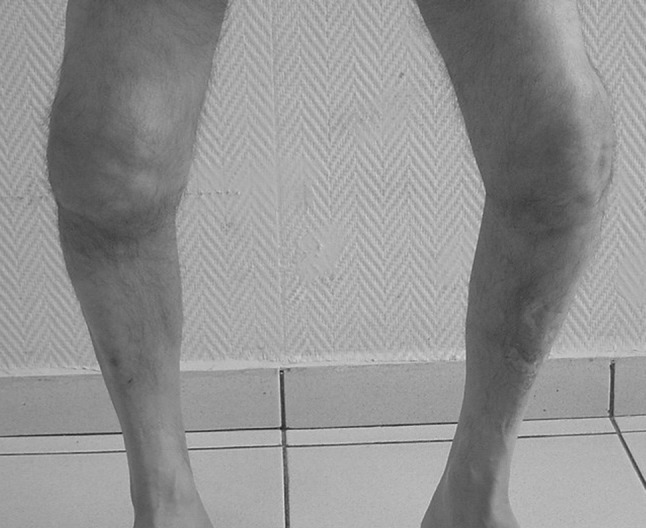
The swelling and the deformity of the knees in weight bearing

His knee pain after activities such as walking, stair climbing, or kneeling first appeared on the right side and 1 month later on the left side. A progressive deformity in weight bearing (Fig. 1) associated with swelling were the major complaints despite relatively painless progress. Upon physical examination, both knees had large effusions and slightly limited range of motion (a range of 5–120° of flexion was possible bilaterally). Mediolateral instability at the level of proximal tibia, not of the knee joint itself, was remarkable on both knees and more striking in weight bearing. Despite the progressive deformity and instability, clinical tests and his gait were relatively painless. Due to the progressive instability of both sides, the patient had a difficulty in walking and had to use crutches. All findings were more prominent on the right side.

The patient had stocking-glove-type paresthesia (decreased sensitivity to pain, temperature, and pressure) typical for all cases with polyneuropathy on both lower limbs particularly below the knee joint and decreased patellar and achilles tendon reflexes. On the anterolateral surface of the middle one-third of the left lower leg, there was old scar tissue secondary to diabetic ulceration. The common femoral arteries, the popliteal arteries, the posterior tibial arteries, and the dorsalis pedis arteries on both lower limbs were palpable on examination and the intensity of the pulse was graded as 3+.

**Fig. 2:**
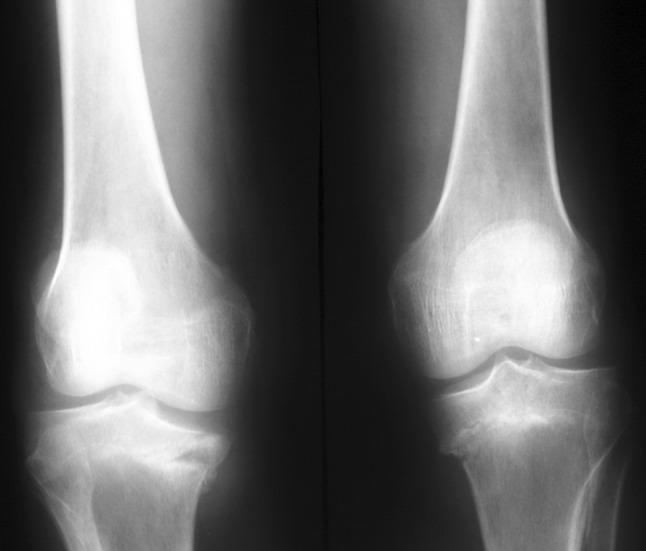
Radiographs of both knees at presentation

**Fig. 3:**
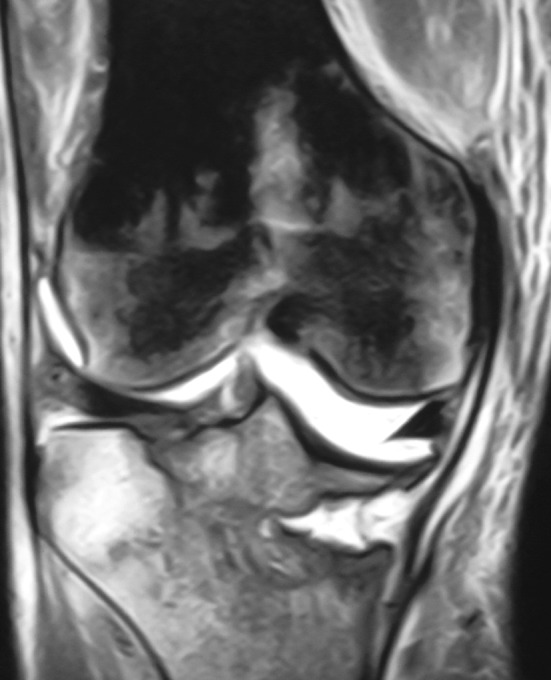
Magnetic resonance imaging (MRI) of the right knee before the operation. The magnetic resonance imaging of both knees showed bilateral tibial plateau fracture on medial side and dense bone marrow edema around, constitutional signal changes on distal femurs, edema on soft tissues around the knee, and effusion on both knees

**Fig. 4:**
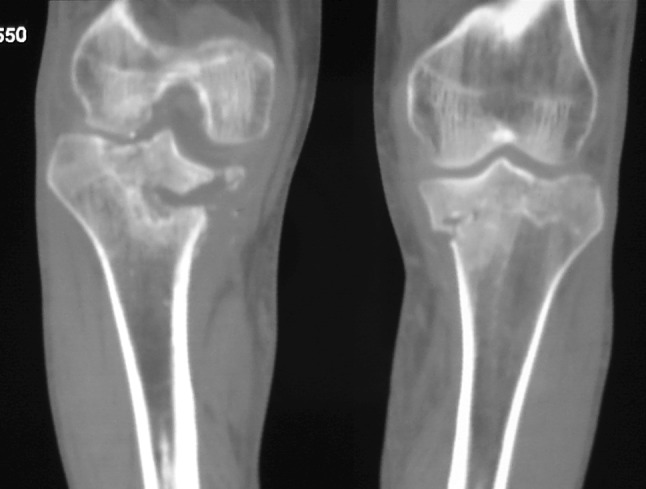
Computed tomography (CT) of both knees. The computed tomography of both knees showed bilateral tibial plateau fracture on medial side, fragmentation, and destruction coalescence

Serial standard X-rays of both knees revealed a progressive insufficiency fracture at the level of medial tibial plateau that was always more severe on the right side (Fig. 2). CT and MRI examinations of both knees delineated a more distinct fracture line, dense bone marrow edema, edema of soft tissues around the knee, and joint effusion that also showed some constitutional signal changes (hypointense marrow signal on all T1 and T2 sequences) on distal femurs (Figs. 3, 4).

The white blood cell counts and CRP of the patient obtained before surgery were normal. MRI findings before surgery were also confirming for ruling out osteomyelitis as a differential diagnosis.

A DEXA analysis of lumbar spine and the right hip region revealed a generalized decrease in bone density. A high-degree sensorio-motor polyneuropathy (diffuse slowing of sensory and motor nerve conduction velocities) has been detected in an EMG examination of both limbs consistent with diabetic neuropathy. Laboratory and ultrasonographic findings disclosed no abnormality of thyroid and parathyroid glands.

The Knee Society knee scores were 49 and 38 points for the left and right knee, respectively, and the function score was 0 (house-bound, impossible stairs, crutches on both sides).

**Fig. 5:**
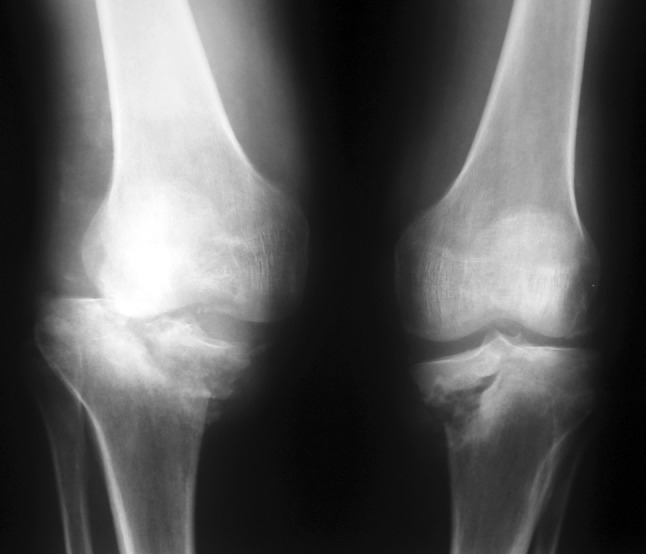
Progression of collapse of the insufficiency fracture and deformity in three months

Our initial method of choice for treatment was conservative, which consisted of protective weight bearing and brace usage. The insufficiency fracture that began from the medial tibial plateau progressed into an intra-articular level just lateral to the tibial eminences. Fragmentation and destruction coalescence also appeared. This destructive progression occurred in a relatively short period of time, only in 3 months from the onset of symptoms, and accentuated the instability (Fig. 5).

Our management with conservative measures failed related to the rapid progression in bone loss, deformity, and instability. In order to stop relentless destruction and to get pain-free and functional knee joints, we changed our treatment modality for replacement surgeries. Since relatively demanding and open-to-complication surgeries for each side, we planned to operate on in two separate sessions. We had to remove all necrotic bone from the tibial plateau and replace them with metallic augments and to use a long-stemmed tibial component. We implanted a current tricompartmental PCL-replacing-type primary prosthesis (NexGen Complete Knee Solution System, Zimmer, Warsaw, IN, USA) with an extension stem and metallic augments on the tibial component. As the amount of resected necrotic bone was more than expected, we had to modify and combine augments to replace the defect, although the prosthesis has not been designed for this type of modification. We did not use any bone graft or bone substituting material. Nor we did use a tumor-resection-type or a custom-made prosthesis to get a more functional and durable outcome. In order to compensate the removal of a large segment and support medial soft tissues, we preferred to use a hinged brace postoperatively.

Immediately after the first surgery on the right side, we encountered a weakness of the ankle and toe extensors. We related this problem to the stretching of the peroneal nerve during extensive surgery. Fortunately, it resolved spontaneously within a week and did not postpone the scheduled surgery for the left side. After a 15-day interval, we performed the similar surgery on the left side. Despite the heel protection, most likely related to the pain insensitivity, a pressure ulcer occurred under the left heel and healed with wound care within 6 weeks after the operation.

**Fig. 6:**
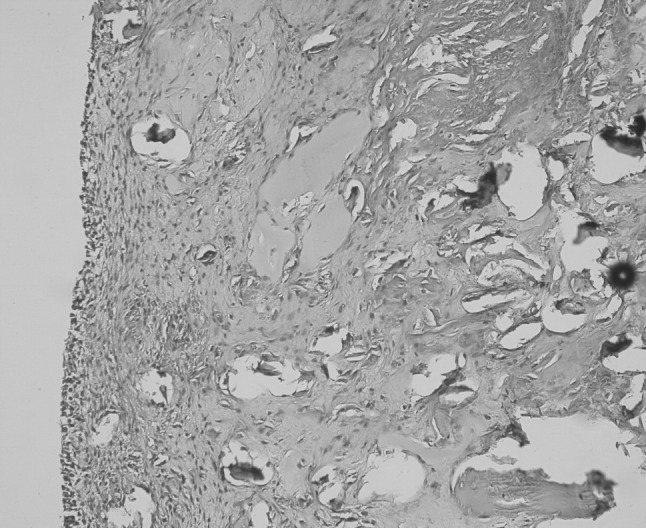
Pathologic examination of tissues revealed severe osteoporosis and severe degenerative changes on joint surfaces. The scarred and chronically inflamed synovium is filled with multiple irregular fragments of bone and cartilage denoting a rapid breakdown of the joint

The histopathological tissue examination was of significant importance in differentiating the neuropathic arthropathy from an isolated insufficiency fracture that was related to chronic mineral imbalance and osteonecrosis. It revealed joint surface cartilage has been destroyed in many areas, and the remaining areas of articular cartilage contained vertical clefts without obvious chondrocyte proliferation. Subchondral bone was highly osteoporotic. There was synovial hyperplasia accompanied by abundant fragments of cartilage and bony debris within the soft tissues around the joints, denoting a rapid breakdown of the joint. Presence of minimal inflammation with mononuclear cells was helpful for differential diagnosis from rapidly destructive inflammatory arthritis, such as rheumatoid arthritis. Osteonecrosis was not found. Considering the clinical findings, the histopathologic changes were accepted to be in accordance with neuropathic arthropathy (Charcot joint) (Fig. 6). Intra-operative cultures did not revealed any microorganism.

**Fig. 7:**
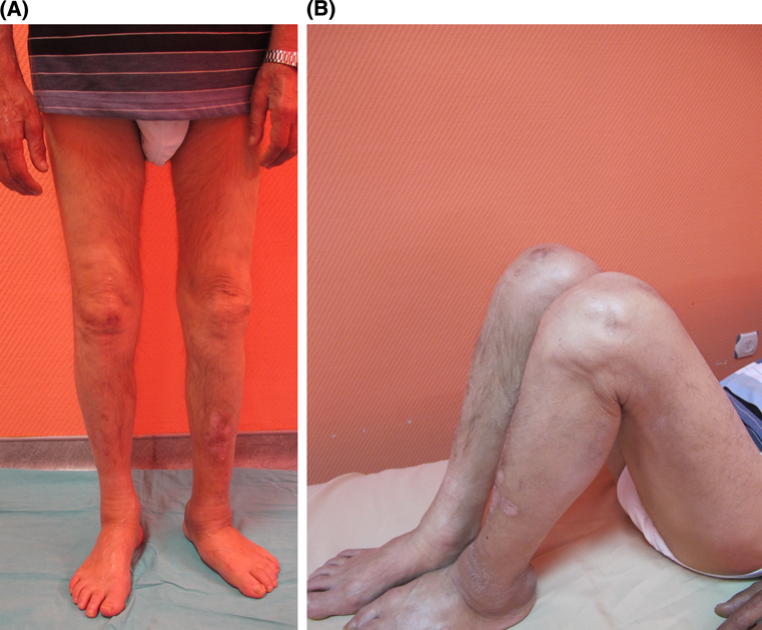
Clinical picture of knees sixty months after the operation. **a** Correction of deformity in weight bearing. **b** 120° of flexion of both sides

**Fig. 8:**
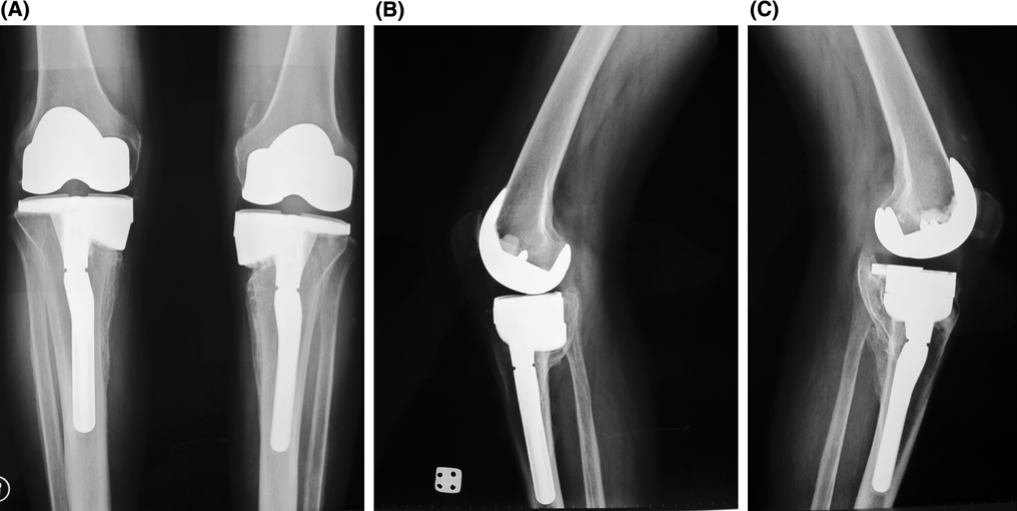
Last control radiographs of both knees. **a** Anteroposterior view of both knees, **b** lateral view of right knee, **c** lateral view of left knee

Three months after the surgeries, we discontinued the brace usage on the right side because no dynamic instability during gait persisted. We followed up the patient regularly up to 5 years. During the latest follow-up at 60th months, the patient could walk without any knee problems. He was brace-free and without any dynamic instability. He was able to flex both knees to 120° and had a full extension (Fig. 7a, b). But his walking capacity was limited related to the Charcot foot on the left side developed in last 2 years. Radiographically, the components of both knees were stable without any sign of loosening (Fig. 8a, b, c). At the latest follow-up, the Knee Society knee and function scores improved significantly. The knee scores increased to 95 and 85 points for the left and right knees, respectively. Function score was only 80 points as the Charcot’s foot-related walking problems limited walking capacity.

Informed consent of the patient was obtained before we prepare this case report.

## Discussion

Neuropathic arthropathy is a progressive joint disease leading to severe deformity, instability, and dysfunction which occurs in the absence of protective neurostimulation. The diminution or absence of nociception resulting in poor joint protection and undetected micro trauma is thought to be responsible for the development of bone destruction and attenuation of ligaments [3]. Although its classical description is related to central nervous system disorders such as tabes dorsalis, currently its association with peripheral nerve lesions, that is diabetic neuropathy, is more and more commonly reported. At the present, diabetes mellitus is considered to be the most common cause of Charcot joint development [4].

The most common symptom of neuroarthropathy in a diabetic patient is a single, painless, swollen, deformed foot without history of trauma. A marked predilection for the tarsometatarsal, tarsal, and ankle joints occurs and the involvement of large weight-bearing joints, such as the knee joint, is rare [5]. Since the early recognition of radiographic abnormalities, prompt treatment may be effective in preventing the later development of serious deformity and disability. It has been recommended to consider diabetic neuroarthropathy in cases with diabetes, peripheral neuropathy, and swollen knees [5].

Our case had some special characteristics that differentiate it from an isolated insufficiency fracture, relatively painless nature onset, and progress of clinical picture, and histological findings like presence of minimal inflammation with mononuclear cells and the absence of the osteonecrosis direct us to the diagnosis of neuroarthropathy [6].

The major distinction between the presented case and those previously reported is the association of two important complications of chronic pancreatitis in this case report: diabetes mellitus and chronic diarrhea. They both may affect bone and joint status. The combined effect of these two factors may be the reason of rapid progression and destruction. Progression in neuropathic arthropathy related to other diseases is generally slow [7], but the disturbance in mineralization related to chronic diarrhea might have accentuated the disease progression.

The high complication rate with all treatment modalities proposed for neuropathic knee joint causes difficulty in developing guidelines for the management. Treatment with conservative measures (bracing, protective weight bearing) may be partly successful only if a very early diagnosis has been established [7]. Unfortunately relatively painless nature of the disease, the common presentation is only in the late stages with marked destruction.

Arthrodesis has been the mainstay of surgical management in the late stages of neuroarthropathy [4]. Although it seems safer and less problematic than arthroplasty, the results are quite variable and it is generally difficult to obtain a solid fusion. In our patient, we did not prefer the arthrodesis as a treatment of choice for two main reasons. Firstly, bilateral knee arthrodesis would be functionally extremely disabling. Secondly, a solid fusion was nearly impossible to obtain in a case with rapid progress, long segment involvement, and severe osteoporosis.

The use of total knee arthroplasty in the management of neuropathic arthropathy is controversial. It has been considered as a contraindication by some for increased risk of infection, periprosthetic fractures, extensor mechanism ruptures, and loosening of components [1, 2]. As for some others, the outcome of total knee arthroplasty in patients with end-stage Charcot’s joints, although somewhat inferior, is not significantly different to that of arthroplasty in other patients [3, 7, 8]. It is no longer considered as an absolute contraindication to arthroplasty [3, 7, 9, 10]. Recommendations for a successful total knee arthroplasty in Charcot’s joints include correct limb alignment and ligamentous balancing, bone grafting and/or custom-augmented prosthesis to repair bony defects, the use of long-stemmed and posterior stabilized components [3, 4, 8, 10]. One of the few articles on arthroplasty use in Charcot’s knees is the only long-term study in neurosyphilis cases. The authors reported satisfactory clinical results in most cases with rotating hinge prostheses after 10- to 22-year follow-up [10].

The experience with arthroplasty in diabetic neuroarthropathy cases is even more limited [3–5, 8, 11]. These case reports describe some successful use of arthroplasty in those cases.

It has also been suggested that rotating-hinged prosthesis should be used for patients with grossly unstable knees, including neuropathic joints [10]. A constrained prosthesis is designated to provide intrinsic stability, but such constraint results in increased stress at the cement-bone and cement-implant interfaces. This may lead to higher rates of early aseptic loosening [1]. They also require the removal of a significant amount of bone not only from tibia, but also from femur, which was not primarily affected by the disease. Furthermore, the success of hinged-type knee prostheses is not much better than that of the more bone preserving procedures.

Although the use of bone grafts or bone substitutes even porous tantalum have been reported previously [11], we did not use any of such materials since the incorporation of new material to the poor-quality host bone would be relatively long and difficult [10].

We performed the replacement surgeries in separate sessions with a short-time interval. We considered each surgery as demanding with indefinite amount of bone to be removed, as well the relatively high risk of complications during the surgery. Simultaneous bilateral replacement would have been even more complicated. After the first surgery, early problems occurred, such as weakness of the ankle and toe extensors. This would delay the surgery on the other side if they were not rapidly reversible. We also thought that a longer interval would increase problems on the opposite side and cause difficulty walking because of the difference in leg lengths.

All surgical procedures, including arthroplasty, have higher complication rates in neuropathic joints than in non-neuropathic arthroplasty cases [1, 3, 9, 10]. This was the case with our patient. Three complications for two knee replacements occurred: mild early mediolateral laxity, temporary weakness of ankle and foot extensors, and pressure ulcer in posterior heel region, always on one side. At the latest visit, the last two problems healed uneventfully but the first one, although minor, persisted. Its long-term effect on the survival of the implant is not foreseen at the moment. Diminished or absence of deep sensation and proprioception as well as the relative painlessness in neuropathic arthropathy make all procedures more complicated. For the success of an arthroplasty procedure in neuropathic joint, a meticulous surgical technique and choice of the proper implant with adequate constraint are not sufficient. In the absence of protective pain sensitivity, all possible complications should be taken into consideration, as well as closely monitored clinical surveillance.

Bilateral staged total knee arthroplasty replacing all fragmented bone material was effective and durable but also open-to-complication option for our case with rapidly progressive and destructive neuroarthropathy.
